# Plants Know Where It Hurts: Root and Shoot Jasmonic Acid Induction Elicit Differential Responses in *Brassica oleracea*

**DOI:** 10.1371/journal.pone.0065502

**Published:** 2013-06-11

**Authors:** Tom O.G. Tytgat, Koen J. F. Verhoeven, Jeroen J. Jansen, Ciska E. Raaijmakers, Tanja Bakx-Schotman, Lauren M. McIntyre, Wim H. van der Putten, Arjen Biere, Nicole M. van Dam

**Affiliations:** 1 Department of Ecogenomics, Institute for Water and Wetland Research, Radboud University Nijmegen, Nijmegen, The Netherlands; 2 Department of Terrestrial Ecology, Netherlands Institute of Ecology, Wageningen, The Netherlands; 3 Department of Chemometrics, Radboud University Nijmegen, Nijmegen, The Netherlands; 4 Department of Molecular Genetics & Microbiology, University of Florida, Gainesville, Florida, United States of America; 5 Laboratory of Nematology, Wageningen University, Wageningen, The Netherlands; BASF Cropdesign, Belgium

## Abstract

Plants respond to herbivore attack by rapidly inducing defenses that are mainly regulated by jasmonic acid (JA). Due to the systemic nature of induced defenses, attack by root herbivores can also result in a shoot response and *vice versa,* causing interactions between above- and belowground herbivores. However, little is known about the molecular mechanisms underlying these interactions. We investigated whether plants respond differently when roots or shoots are induced. We mimicked herbivore attack by applying JA to the roots or shoots of *Brassica oleracea* and analyzed molecular and chemical responses in both organs. In shoots, an immediate and massive change in primary and secondary metabolism was observed. In roots, the JA-induced response was less extensive and qualitatively different from that in the shoots. Strikingly, in both roots and shoots we also observed differential responses in primary metabolism, development as well as defense specific traits depending on whether the JA induction had been below- or aboveground. We conclude that the JA response is not only tissue-specific but also dependent on the organ that was induced. Already very early in the JA signaling pathway the differential response was observed. This indicates that both organs have a different JA signaling cascade, and that the signal eliciting systemic responses contains information about the site of induction, thus providing plants with a mechanism to tailor their responses specifically to the organ that is damaged.

## Introduction

In their natural environment, plants are subject to attacks by a wide variety of root and shoot herbivores. Plants respond to above- or belowground herbivore feeding by increasing the production of defense compounds [1], [2]. These induced responses can contribute to plant resistance by reducing herbivore performance or by attracting the herbivores’ enemies, e.g. predators and parasitoids, to the plant [3], [4], [5]. Jasmonic acid (JA) is by far the most well studied phytohormone involved in herbivore-induced responses in plants [6], [7], [8]. JA is synthesized from alpha-linolenic acid by a series of lipoxygenase (LOX) enzymes [8]. The biosynthetic enzymes - such as LOX2, AOS, OPLC1- are well characterized, and genes coding for these enzymes are known to be up regulated when plants are challenged by wounding, chewing herbivores or necrotrophic pathogens [7], [9], [10], [11], [12]. Upon herbivore damage, JA levels increase within seconds to minutes [7], [13].

Local JA production or ectopical JA application also induces systemic responses in undamaged or untreated plant parts. Systemic JA induction of defense responses also occurs from roots to shoots and *vice versa*, thereby affecting the performance of herbivores and natural enemies in the above- and belowground compartment [1], [14], [15], [16], [17], [18]. In addition to the production of defense compounds, JA also causes the re-allocation of primary metabolites between roots and shoots [19], [20], [21]. It is postulated that the reallocation of primary metabolites is the signature of metabolic reprogramming needed to enhance plant tolerance to herbivory. Plants attacked by shoot herbivores may benefit by storing their resources in the roots and re-grow the lost leaf tissue from this pool after aboveground herbivory has stopped [22]. Induction of defensive compounds and reallocation of primary compounds within the plant thus reflect different plant strategies to survive the damaging effects of herbivores [23].

Compared to what is known about JA-induced responses in shoots, we know relatively little about the role of the JA signaling pathway in local and systemic root-induced responses [15], [24]. Given the different physiological functions of roots and shoots, wounding or infection of either organ will likely pose different challenges to the plant in order to minimize the effect of herbivory on its performance. Recent studies indeed have shown that the induction of various JA-responsive defensive compounds, such as glucosinolates, phenylpropanoids and terpenes, differ depending on whether the JA is applied to the shoots or to the roots of *Brassica* plants [2], [25], [26], [27]. Similar findings have been reported in studies using real aboveground and belowground herbivores to induce the plant [15], [16], [28]. We therefore hypothesize that plants are able to detect which organ is attacked and that the local and systemic response to JA will largely depend on the tissue that is induced. To test this hypothesis, we applied JA to either the roots or the shoots of *Brassica oleracea* and analyzed the transcriptome profiles in both organs with the genome-wide *Arabidopsis thaliana* 70 bp oligo chips. The *Brassica* genus is closely related to *A. thaliana*, which is reflected by an average 87% sequence homology in the coding regions of homologous genes (http://ukcrop.net/brassica.html). Moreover, the suitability of these long oligo *A. thaliana* arrays to analyze gene expression in *Brassica* species has been demonstrated before [29], [30], [31]. Depending on the herbivore species, the plant responses to herbivore feeding are controlled by a mix of several hormonal pathways, whereby the JA pathway is the main signaling pathway that integrates the perceived information at the site of attack into the defense response [4]. Hence, although ectopic JA application does not completely mimic herbivore feeding, JA or its methyl-ester, methyljasmonate, is often used to facilitate the quantitative and qualitative analysis of herbivore-induced plant responses governed by JA signaling [32], [33], [34], [35]. This is specifically relevant when comparing above- and belowground JA-induced responses, as insect herbivores that feed on both root and shoot tissue in the same life stage are rare. Here, we investigated the effect of organ specific JA induction on gene expression in primary and secondary metabolism, plant development and the early JA signaling cascade. Additionally, we analyzed sugar and amino acid levels in the roots and the shoots of the same plants.

## Materials and Methods

### Plant Growth and JA Induction

Seeds from a wild accession (The Netherlands) of *Brassica oleracea* were germinated on glass beads and water for one week, and the seedlings were transferred to individual 1.3 L pots containing sterilized plain river sand. The pots were maintained in a greenhouse at 21°C (day) and 16°C (night), room humidity 60%. Natural daylight was supplemented with sodium lamps to maintain the minimum PAR at 225 µmol.m^−2^.s^−1^ with a photoperiod of 16:8 (L:D). Twice, and later three times per week, the plants were provided with sufficient half-strength Hoagland solution with a doubled P-content to maintain the water percentage in the pots at 14% w/w [2]. Thirty-three days after the seedlings were transferred to the pots, 270 plants of equal size and appearance were selected. By that time, the plants had on average 9 true leaves, 2.2 (±0.5 s.d.) g dry root mass and 3.8 (±0.1) g dry shoot mass (biomass data obtained from five representative plants that did not enter the induction experiment). The plants were assigned to one of the following three treatment groups: (1) SJA, 500 µg JA (Sigma, St Louis, MO, USA) applied to two fully expanded leaves in 0.250 ml 0.1% Triton in water (pH = 3.3); (2) RJA, 500 µg JA in 10 ml 0.1% Triton (pH = 4.2) applied with a plastic syringe to the sand surrounding the root; (3) CON, equal amounts of acidic (HCl) 0.1% Triton in water (pH = 3.7) applied to roots and shoots as the JA treated plants. Similarly, SJA and RJA plants received acidic water solution to the untreated organ [36]. At 6, 18 and 30 h after JA application, 30 plants (3 pools of 10 plants) of each treatment group were harvested. Leaf samples were taken by punching three leaf discs (9 mm diameter) from both the third and the second youngest leaves of each plant. These leaves were one or two ontogenetic positions younger than the JA-treated leaves. The six discs of 10 individual plants were pooled to obtain one biological replicate. The roots were cleaned with water to remove the sand, and to obtain a representative root sample, three sub-samples were taken equidistantly over the length of the root. The root samples were pooled per 10 plants. Because roots and shoots were collected separately, this resulted in 54 samples (3 time points×3 treatments×3 pools×2 organs) in total.

### Microarray Analysis

Total RNA was isolated with Trizol® Reagent (Invitrogen Corp., California, USA) and further purified on RNeasy Mini Spin Columns (Qiagen GmbH, Hilden, Germany). To allow all possible comparisons between treatment groups, a single color hybridization was performed on the 29,000 element Arabidopsis 70-mer Oligonucleotide Microarrays based on the Qiagen-Operon Arabidopsis Genome Array Ready Oligo Set (AROS) Version 3.0. Hybridization and scanning of the microarrays was performed by the Microarray Hybridization and Analysis Services at the University of Arizona, USA, following their standard procedures (see http://ag.arizona.edu/microarray/). Spot intensities were determined using ImaGene® 7.0 software (BioDiscovery, El Segundo, CA, USA), and transcript abundance was estimated as the natural log of the spot mean minus the mean of the local background. Transcript levels were normalized by centering to the median value of all genes on the slide. We analyzed the data separately for each combination of organ and time point (thus splitting the experiment into six subexperiments) and all data checks and analyses described below were carried out separately for each of the six subsets of slides. For each slide we set a threshold for spot detection at the 95^th^ percentile of the distribution of negative controls. The analyses only included those probes for which at least two of the three replicates were above the detection threshold in each of the SJA, RJA and CON treatments. We further excluded all probes from the analysis whose intensity scores in all treatments and in all replicates were within the lowest quartile of the distribution of intensity scores of all genes on the slide. These low-expression probes could include *A. thaliana* genes that are either absent in *Brassica* or that are too dissimilar between both species to permit effective hybridization. One slide (root tissue, CON treatment) was discarded as it showed overall low gene expression values, and visual inspection of residuals indicated that the statistical model did not fit well to data of this slide. Of the 29109 *Arabidopsis* gene probes present on the slide we included 24007 in our analysis.

ANOVA models were fitted for each probe to test effects of treatments on transcript levels using SAS 9.1 software (The SAS Institute, Cary NC). Residuals were tested for normality (Shapiro-Wilk test) and were examined visually for homogeneity of variances, confirming overall good conformation to standard ANOVA model assumptions. We interpreted *P*-values of two contrasts: control plants versus root induced plants, and control plants versus shoot induced plants. Across the total set of *P*-values resulting from these two contrasts we set an FDR threshold of 10% to declare *P*-values significant [37]. Further limiting the number of genes by lowering the threshold for significance (FDR corrected *P*-value <0.05) resulted in a gene list that was more difficult to interpret biologically.

All gene annotations were done according to the *Arabidopsis* TAIR 9 January 2010 version, with some modifications based on more recent publications. Heat map construction and clustering of genes was done with the MultiExperiment Viewer software package from the TM4 microarray software suite [38]. Average fold change analysis of gene expression per gene functional classification bin was done with the PageMan software package [39]. Functional classification of genes per bin was done with the Classification SuperViewer Tool (http://bar.utoronto.ca/ntools/cgi-bin/ntools_classification_superviewer.cgi).

To fulfill the MIAME requirements, all microarray data were deposited in the Gene Expression Omnibus (GEO) database (http://www.ncbi.nlm.nih.gov/geo/) and received the series record number GSE38784.

### RT-qPCR Analysis

To verify the gene expression profiles obtained by microarray analysis, an RT-qPCR analysis was performed on separate batch of total RNA from the same pool of biological samples as described above. For primer design, orthologous sequences of the respective *A. thaliana* gene coding sequence were collected from all *Brassica* ssp. sequences available in GenBank. Primers were designed on conserved stretches within the *Brassica* orthologous sequences, and the specificity was verified by sequencing of the amplification product. The primer sequences with corresponding orthologous *A. thaliana* AGI locus are in Table S1. For each sample, 1 µg of total RNA was reverse transcribed into cDNA with oligo(dT)_20_ and SuperScript™ III Reverse Transcriptase (Invitrogen Corp., California, USA) according to the manufacturer’s instructions. Subsequently, all samples were diluted five-fold with water. The qPCR amplification mix consisted of: 5 µl diluted 1^st^ strand cDNA, 0.2 µl forward primer (10 µM), 0.2 µl reverse primer (10 µM), 12.5 µl qPCR SYBR green mix (Thermo Scientific, Waltham, MA, USA), and 7.1 µl H_2_O. The qPCR was performed on the Rotor-Gene 3000 (Corbett Research, Sydney, Australia) according to the following protocol: an initial denaturation for 15 min at 95°C, followed by 45 cycles of 30 s at 95°C, 30 s at 58°C, 30 s at 72°C. The relative expression levels of the target genes were calculated by normalization with the expression of the two reference genes *GAPC2* and *PP2A* [40]. Fold changes in gene expression levels were calculated by dividing the mean normalized expression [41] of the treatment group by those of the control.

### Chemical Analysis

A separate batch of leaf discs taken as above was used to analyze glucosinolate levels at 6, 18 and 30 h after induction. The discs were freeze-dried, ground, extracted and analyzed on reversed phase HPLC as described in [36]. Sugar and amino acid analyses were performed on leaf and root samples from a different set of experiments using the same seed batch and JA treatment groups [25]. For this experiment, the entire shoots and roots were harvested 1, 3, 7 and 14 days after treatment, freeze-dried, extracted and analyzed on an ion-exchange HPLC with electrochemical detector as described previously [36]. The statistical significance of the average change in amino acid and sugar concentrations between the control and SJA or RJA samples was determined by a t-test assuming unequal variances (n = 10, p<0.05).

## Results and Discussion

### Gene Expression is Determined by Time Point, Tissue and Site of JA Induction

JA was applied either to the shoots (Shoot JA Application, SJA) or to the roots (Root JA Application, RJA), whereas control (CON) plants received a mock treatment. Systemic (untreated) leaves and the whole root system were harvested 6, 18 and 30 h after JA induction. To validate the gene expression patterns obtained by microarray analysis, we performed an RT-qPCR analysis for five genes involved in defense signaling and secondary metabolite production and compared the results to those of the microarray (Figure S1). Even though small differences were found in the strength of the response after JA treatment compared to control, the overall patterns were very similar.

For each time point and tissue, the log_2_ of the fold change in gene expression between RJA or SJA and the control treatment was calculated, resulting in twelve different transcriptome profiles. To analyze differences and similarities between all transcriptome profiles, correlation coefficients between the fold changes for every gene were calculated (Table 1). For identical tissues and sites of induction, the correlation coefficient between different time points was 0.35 at most, but more often much less and sometimes even negative. Correlation coefficients for the transcriptome profiles of the same time points but of different tissues were, depending on the site of induction, quite different and became very low at the latest time point (varying from 0.26 to 0.66 at 6 h, but only from 0.06 to 0.30 at 30 h). Correlation coefficients between transcriptome profiles of the same tissue and time point that only differed in the site of induction were on average about 0.65, but also lower at 30 h than at 6 h. This indicates that for all treatment groups, the transcriptome profiles were changing very rapidly in time and were determined by tissue as well as the site of JA induction.

**Table 1:** The correlation coefficients of the transcriptome profiles of all treatment groups.

			Roots						Shoots					
			RJA			SJA			RJA			SJA		
			6	18	30	6	18	30	6	18	30	6	18	30
**Roots**	**RJA**	**6**	1	0.20	0.22	0.61	0.18	0.12	0.26	0.09	−0.12	0.39	0.04	0.17
		**18**		1	0.27	0.05	0.62	−0.05	0.32	0.32	−0.06	0.10	0.37	−0.04
		**30**			1	0.23	0.02	0.60	0.18	0.19	0.06	0.19	−0.06	0.30
	**SJA**	**6**				1	0.25	0.20	0.43	0.10	−0.26	0.66	−0.06	0.19
		**18**					1	−0.20	0.32	0.10	−0.15	0.25	0.44	−0.03
		**30**						1	0.04	0.16	0.06	0.15	−0.14	0.30
**Shoots**	**RJA**	**6**							1	0.35	−0.06	0.77	0.27	0.31
		**18**								1	0.23	0.31	0.69	0.32
		**30**									1	−0.17	0.28	0.58
	**SJA**	**6**										1	0.18	0.30
		**18**											1	0.22
		**30**												1
**Total no sign.**			128	1451	685	0	2442	205	451	568	1579	5901	1628	832

For calculation of the correlation coefficients, we used the fold changes in expression compared to control treatment for all measured genes after RJA and SJA. The number of genes that were statistically significantly up or down regulated compared CON (ANOVA with FDR corrected *P*-value <0.1) are shown at the bottom of the table.

The number of genes that were significantly up or down regulated after RJA or SJA (ANOVA, p<0.1 after FDR correction) also showed considerable differences depending on the tissue as well as the site of JA induction (Table 1). Already 6 h after SJA, the shoots showed a massive change in gene expression comprising 5901 genes. Thereafter, the number of significantly induced or repressed genes steadily declined. Compared to these quick and massive responses to SJA, shoot responses to RJA were much slower and less extensive, reaching a peak only after 30 h. In the roots, a much weaker response was observed to RJA at 6 h, whereas none of the genes in this tissue significantly responded to SJA at that time point (Table 1). Root responses to both RJA and SJA peaked after 18 h and then declined again. Because the tissue specificity of the JA response has already been very well demonstrated [12], our further analysis focuses on the effect of the site of JA application.

### Gene Functional Classification Bin Analysis

**Figure 1:**
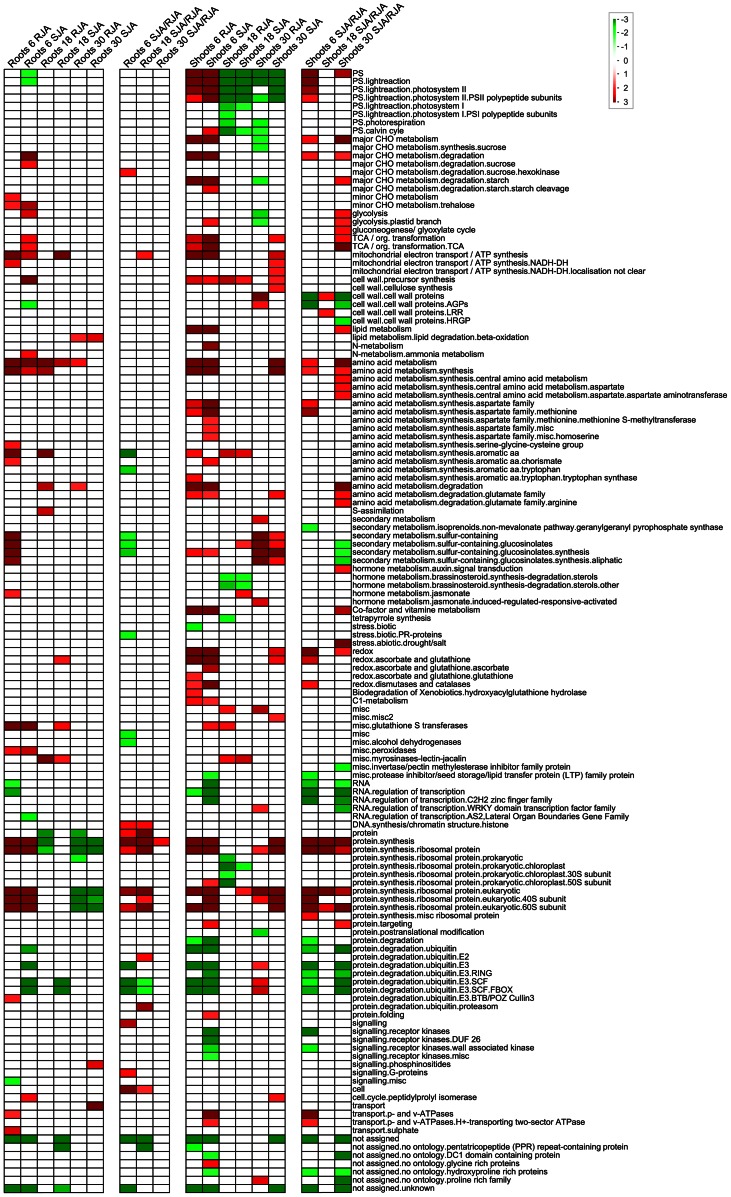
Average fold change analysis per gene functional classification bin. The average fold change per bin was analyzed with PageMan on all measured genes in roots and shoots at 6, 18 and 30 h after JA treatment. The shoots showed a more extensive JA response than the roots. In roots and shoots, the Amino Acid and Protein Synthesis bins were strongly induced, indicating large metabolic changes. In both tissues, several bins were only affected by one of the treatments. Fold changes were either RJA/CON, SJA/CON or SJA/RJA. Bins showing a significantly higher or lower average fold change compared to all other bins are highlighted with a red or green gradient, respectively, while bins that were not significantly affected in any of the treatment groups were omitted from the table (Wilcoxon test with Benjamini-Hochberg multiple testing correction).

To analyze the JA response after the different treatments, gene functional classification bins showing a significantly different mean fold change compared to all other bins were identified by a PageMan analysis (Figure 1) [39]. Because we specifically aimed to identify the differences between a RJA and SJA, also the log_2_ of the fold changes of SJA over RJA were considered in addition to the fold changes of RJA or SJA over CON. Analysis of the SJA/CON and RJA/CON fold changes showed that shoots overall responded more extensively to JA treatment than roots (Figure 1). Nevertheless, in both tissues a strong response was observed for genes in the primary (amino acid synthesis, protein synthesis, major CHO metabolism, glycolysis, TCA cycle, mitochondrial electron transport, lipid metabolism, cell wall precursor) and secondary (glucosinolate synthesis and isoprenoids) metabolism bins, indicating a major metabolic reprogramming. Specific for the shoots was a strong induction of genes in the photosynthesis bin at 6 h, whereupon these genes were strongly repressed at 18 and 30 h.

When comparing the RJA/CON with the SJA/CON fold changes, several bins were significantly affected by only one of the treatments. For instance in the shoots, the genes in the bins for the Calvin cycle, the plastid branch of glycolysis, N-metabolism, and chorismate synthesis were significantly induced only after SJA. In the SJA/RJA fold change analysis most of these bins did not show a significant difference, which indicates that SJA and RJA have the same direction of response but with different strengths. However, a few bins showed significant differences for both the JA/CON contrast and the SJA/RJA contrast. For instance, in shoots, the major CHO (carbohydrate) and amino acid degradation bins were specifically more affected by SJA, while genes in the cell wall protein and the glucosinolate bins responded more strongly to RJA (Figure 1). In roots, RJA significantly induced the bins for aromatic amino acid synthesis and glucosinolate synthesis more than SJA.

### JA-induced Changes in Sugars and Amino Acids

**Figure 2:**
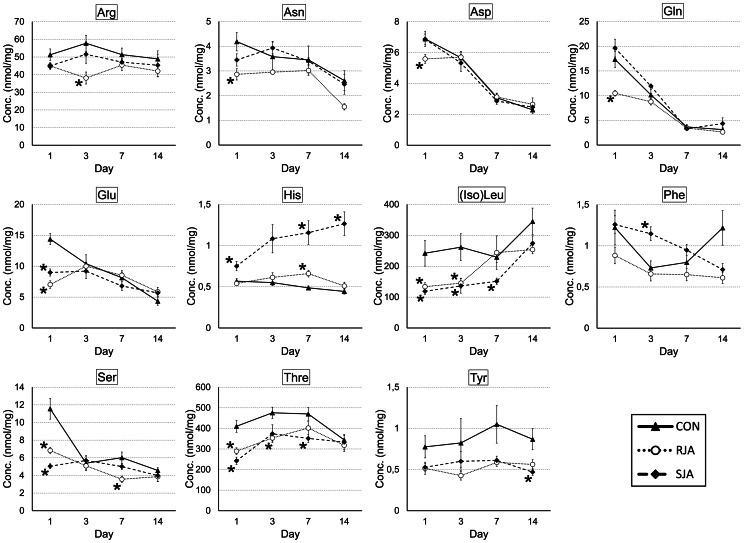
Amino acid concentrations in the shoots. Amino acid concentrations were measured by HPLC in the shoots after RJA, SJA and CON at day 1, 3, 7 and 14. The concentration of almost all amino acids was affected by JA treatment, whereby some (Arg, Asp, Gln, His, Phe) responded differently to RJA than SJA. Concentrations are expressed in nmol/mg dry plant material after RJA (dotted line, open circles), SJA (dashed line, squares) or control treatment (solid line, triangles). Error bars represent standard errors. Samples with a significantly different concentration compared to control are marked with an asterisk (p-value <0.05, t-test independent samples assuming unequal variances).

**Figure 3:**
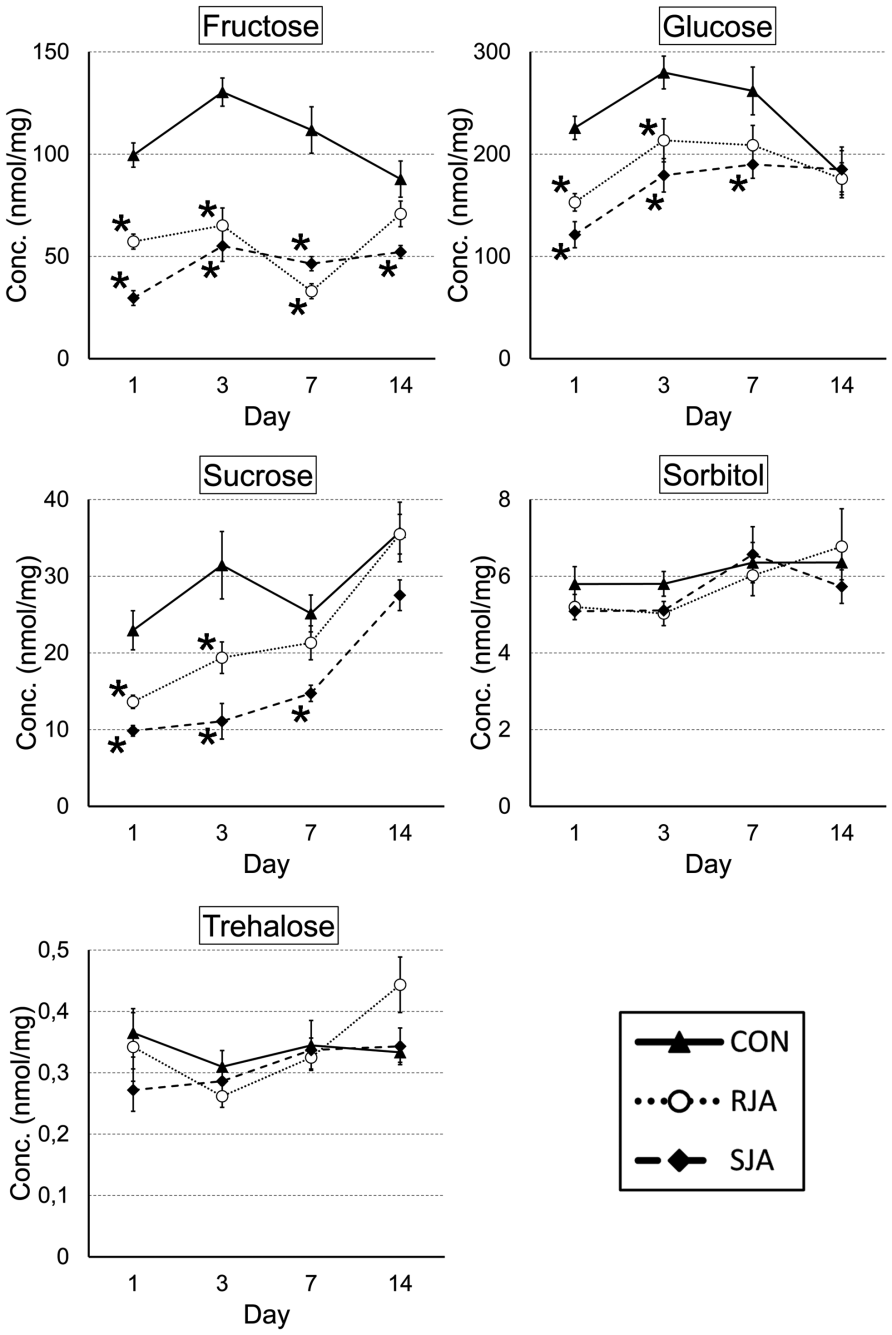
Sugar concentrations in the shoots. Sugar concentrations were measured by HPLC in the shoots after RJA, SJA and CON at day 1, 3, 7 and 14 after JA application. RJA and SJA resulted in a decreased concentration of sucrose, glucose as well as fructose at day 1, 3 and 7. Concentrations are expressed in nmol/mg dry plant material. RJA, dotted line and open circles; SJA, dashed line and squares; CON; solid line and triangles. Error bars represent standard errors. Samples with a significantly different concentration compared to control are marked with an asterisk (p-value <0.05, t-test independent samples assuming unequal variances).

Given the contrasting responses after RJA and SJA of genes involved in primary metabolism, we measured the amino acid and sugar content in roots and shoots harvested 1, 3, 7 and 14 days after JA treatment using HPLC. As for the gene expression patterns (Figure 1), the effect of JA treatment on sugar and amino acid concentration was much larger in the shoots than in the roots (Figure 2, 3, S2, S3). Especially the concentrations of threonine, (iso)leucine, serine and glutamate were significantly reduced in the shoots after both RJA and SJA at day 1. Moreover, RJA caused a significant reduction in glutamine, asparagine, and aspartate concentration at day 1 and of arginine at day 3. In contrast, SJA treatment significantly increased histidine concentrations at almost all time points. In roots, isoleucine showed the strongest response and decreased after RJA and SJA at day 1 and 3 (Figure S2). Glutamate and threonine decreased after RJA and SJA, respectively.

In the shoots, a significant reduction was observed for fructose, glucose and sucrose levels from day 1 until day 7 after both RJA and SJA, whereby the effect was stronger after SJA (Figure 3). In the roots, sucrose levels were significantly decreased 1 and 3 days after RJA and SJA, while fructose levels were only reduced 1 day after RJA, and glucose levels 3 days after SJA (Figure S3). In neither tissue significant changes in sorbitol or trehalose concentrations were found (Figure 3, S3). A detailed analysis of the expression of the genes belonging to the major CHO bin demonstrated that in roots mainly sucrose metabolism was affected (Figure S4A), while in shoots starch degradation was altered (Figure S4B). In RJA treated roots a strong induction of several sucrose transporters (*SUC1, SUC5, SUC7*) was observed, indicating an increased sucrose loading into the phloem, which was not seen after SJA treatment. *Cytosolic Invertase 1* (*CINV1*), encoding a key enzyme in sucrose degradation, was highly up regulated in RJA treated roots and to a lesser extent in SJA treated roots. In the shoots, *Isoamylase 3,* encoding a starch degrading enzyme, showed an almost 4 fold higher repression after RJA than SJA in at 18 h (Table S2). In conclusion, JA application results in a significant shift in primary metabolism, which is visible at the molecular as well as phenotypic level in roots and shoots. In both tissues carbohydrates levels decreased despite enhanced gene expression of sugar transporters, suggesting that other metabolic processes are a sink for the sugars that are released by sucrose and starch degradation.

### Genes with a Large Difference in Expression after RJA versus SJA Treatment

To obtain a more detailed view on which genes exactly responded differentially to SJA versus RJA, we filtered out all genes that showed a significant JA response compared to CON and had at least a three-fold change between RJA and SJA at one of the time points. In total, we found 411 genes that met this criterion (see Table S2 for expression levels of all genes ordered according to the functional classification bins). A functional categorization of the genes was performed with the Classification SuperViewer Tool (Table 2). This analysis confirmed that genes in both primary and secondary metabolism specifically responded depending on where the JA was applied.

**Table 2:** The number of different genes in each functional classification bin showing at least a 3-fold difference in expression after RJA versus SJA in the roots, shoots or in total in both tissues.

Functional classification bin	total	roots	shoots
Co-factor and vitamine metabolism	1	0	1
DNA	8	3	8
OPP	2	1	1
PS	4	1	3
RNA	47	24	33
TCA/org. transformation	1	0	1
amino acid metabolism	3	3	1
Cell	14	10	10
cell wall	8	4	4
development	20	14	12
hormone metabolism	15	12	8
lipid metabolism	12	6	6
major CHO metabolism	1	0	1
metal handling	2	1	2
minor CHO metabolism	4	2	2
miscellaneous	35	20	19
mitochondrial electron transport/ATP synthesis	1	1	1
not assigned	127	67	82
nucleotide metabolism	3	2	1
Protein	41	25	23
Redox	5	2	5
secondary metabolism	11	7	4
signalling	17	11	9
Stress	21	7	15
tetrapyrrole synthesis	1	0	1
transport	15	7	12

### Plant Development

JA is known to control not only defense responses but also several developmental processes such as root growth, pollen development, senescence and fruit ripening [6]. This indicates that the regulation of plant defense responses and developmental processes are highly integrated [42]. Among the genes showing an at least three-fold difference in expression after RJA versus SJA we found several that are involved in developmental processes. For instance, *EFS* (*Early Flowering in Short days*), a histone methyltransferase that epigenetically controls several processes related to flower development [43], [44] had a higher expression at 18 h after SJA than RJA in roots as well as shoots (Table S2). *SPA1,* which is involved in the regulation of circadian clock and photoperiodism [45], on the other hand showed a much higher expression in the roots after RJA than SJA at all three time points. *VSP2*, encoding a vegetative storage protein, showed a much higher expression after RJA than SJA in the shoots at 18 and 30 h (Table S2). *VSP2* is also a commonly used marker gene for JA induction. The fact that this gene is induced stronger after RJA than SJA in the shoots is a strong indication that the shoot response after RJA is not simply a diluted SJA local response. Interestingly, in the roots the expression of *VSP2* did not respond to the JA treatment, which indicates that *VSP2* is not a good marker gene for JA responses in the roots. These results suggest that plants not only adapt their metabolism, but also their development specifically to which tissue is attacked. It is conceivable that this specificity is a functional response, as root damage has a different effect on plant survival than leaf wounding, and therefore elicits different defense responses as well as different modifications of the developmental program.

### Glucosinolate Biosynthesis

**Figure 4:**
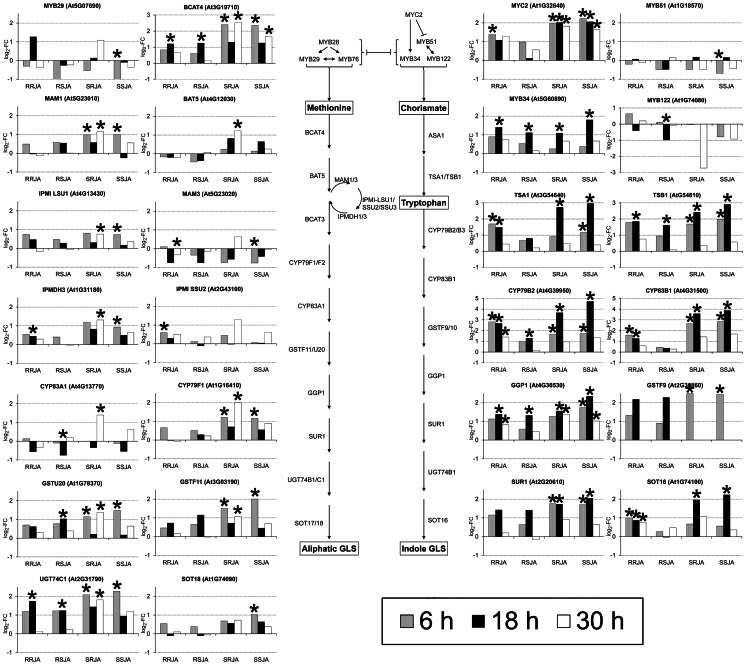
Gene expression in the glucosinolate synthesis pathway. The JA response of the genes involved in glucosinolate synthesis was weaker in the roots than in the shoots. In the shoots, RJA resulted in a stronger induction of several transcription factors and enzymes involved in the biosynthesis of aliphatic glucosinolates than SJA. In contrast, for the indole glucosinolate pathway, SJA lead to a stronger induction of the involved genes than RJA. Histograms represent the log_2_ of the fold changes in expression after RJA or SJA compared CON for all genes that were significantly affected in at least one of the treatment groups (ANOVA, FDR corrected *P*-value <0.1). Samples showing a significantly different expression compared to CON are marked with an asterisk. RRJA, Root tissue RJA treatment; RSJA Root tissue SJA treatment, SRJA, Shoot tissue RJA treatment, SSJA, Shoot tissue SJA treatment.

In both organs, genes coding for the production of sulfur-containing glucosinolates were the most prominent involved in secondary metabolism responding to JA treatment (Figure 1). As observed before at the phenotypic level in several *Brassica* species [46], the JA-induced response of glucosinolate synthesis genes was weaker in the roots than in shoots (Figure 4). However, roots generally have higher constitutive glucosinolate levels than shoots, and it has been suggested that possibly in response to the higher chances of pathogen or herbivore attack belowground, for roots a constitutive defense is more optimal than an induced defense [46], [47]. A detailed analysis of expression patterns in the glucosinolate pathway in shoots and roots showed that several other genes in the aliphatic as well the indole glucosinolate synthesis pathway were differentially induced after RJA and SJA (Figure 4). The gene encoding the transcription factor Myb29 that controls the aliphatic glucosinolate synthesis was slightly up regulated after RJA but repressed after SJA. Moreover, several genes encoding enzymes involved in the aliphatic glucosinolate pathway (*BCAT4, BAT5, IPMDH3, CYP79F1, and CYP83A1*) were also more strongly induced in shoot tissues after RJA than after SJA at all three time points (Figure 4, left panel). In contrast, SJA caused a stronger induction of the *MYB34* transcription factor and the enzymes *TSA1*, *TSB1*, and *CYP79B2* (three-fold, Table S2), all involved in indole glucosinolate biosynthesis (Figure 4, right panel). This indicates that after RJA mainly aliphatic glucosinolates, and after SJA mainly indole glucosinolates are induced. Previous results with several *Brassica* species indeed showed consistently higher concentrations of indole glucosinolates in their shoots seven days only after SJA [2], [46], [48]. Aliphatic glucosinolates levels in the shoots, on the other hand, increased only after RJA [2], [36]. Similar to what was observed for the VSP2 induced response, we could confirm that the difference in glucosinolate response after RJA and SJA is not merely an effect of a dilution of the signal. In *B. rapa* we showed that even when the amount of JA applied to the roots is tripled, only SJA can increase indole glucosinolate levels in the shoots seven days after application, whereas RJA cannot (Figure S5). HPLC analysis of root and shoot tissues of the same plants used for expression profiling showed no quantitative differences in glucosinolate concentrations between the treatments after 6 to 30 h (data not shown). Most likely, the time was too short for sufficient accumulation of glucosinolates. Generally, three to seven days are needed to find significant increases in glucosinolates after JA application in *Brassica* plants [49].

### Terpenoids

**Figure 5:**
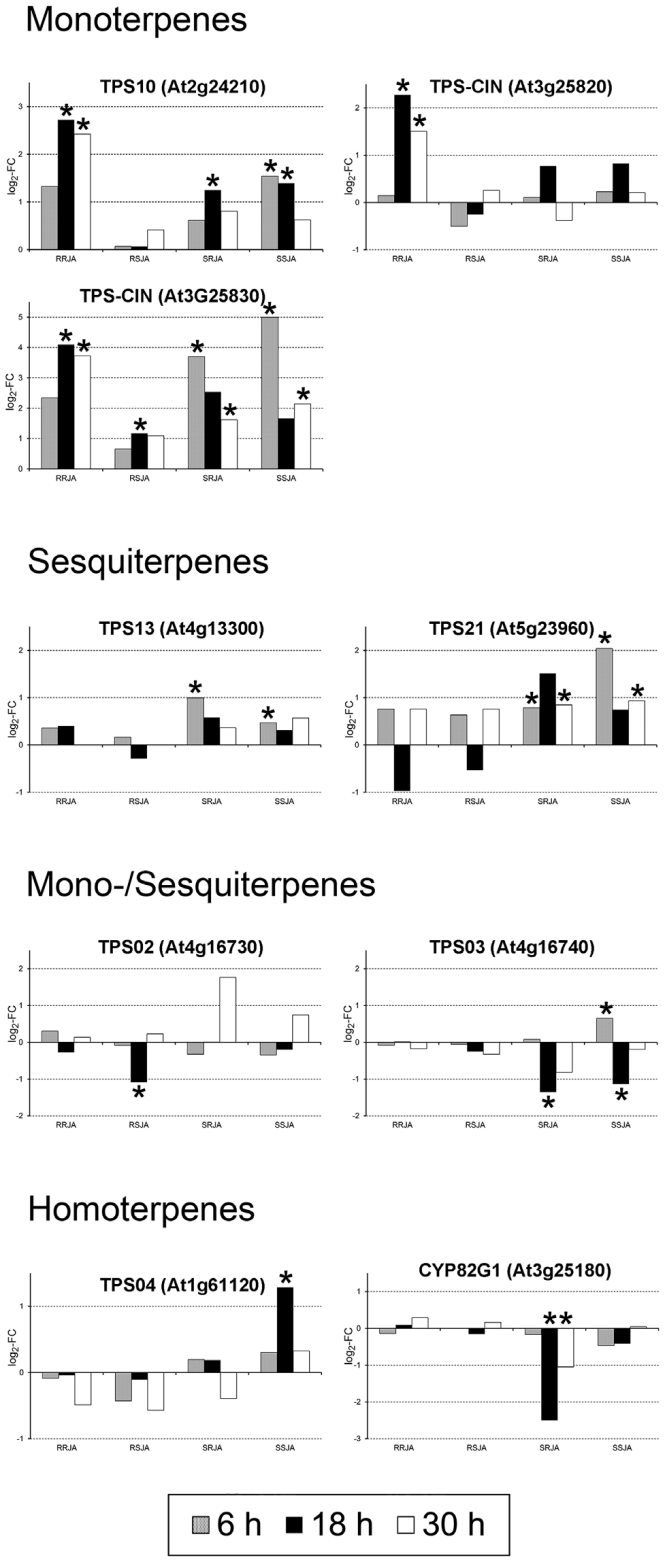
Volatile terpene biosynthesis genes respond differently to RJA versus SJA Treatment. In the roots, RJA strongly induced the monoterpene synthesis genes, while SJA did not. In the shoots, the homoterpene synthesis gene *TPS04* was only induced by SJA, while RJA even significantly repressed the homoterpene synthesis gene *CYP82G1*. Expression is shown as log_2_ of fold changes RJA or SJA compared to CON. Samples in which the gene showed a significantly altered expression compared to CON are marked with an asterisk (ANOVA, FDR corrected *P*-value <0.1). RRJA, Root tissue RJA treatment; RSJA Root tissue SJA treatment; SRJA, Shoot tissue RJA treatment; SSJA, Shoot tissue SJA treatment.

In the secondary metabolism bin, four genes (*CYP82G1*, *TPS10*, and 2 *TPS-CIN*) involved in terpenoids synthesis were differentially regulated by RJA and SJA (Table S2). A detailed analysis of all significantly induced genes involved in the synthesis of homo-, mono- and sesquiterpenes showed that in the roots, RJA led to a strong induction of the monoterpene synthesis genes *TPS10* and both *TPS-CIN* genes, while these genes did not respond to SJA (Figure 5). No significant responses were observed for genes involved in sesquiterpene or homoterpene synthesis in the roots. In the shoots, SJA elicited the induction of monoterpene synthesis genes (*TPS10* and one of the *TPS-CIN*), the sesquiterpene synthesis gene *TPS21*, and the homoterpene synthesis gene *TPS04*. After RJA, the monoterpene synthesis genes *TPS10* and one of the *TPS-CIN* were induced in the shoots, as well as the sesquiterpene genes *TPS13* and *TPS21*. In contrast, the *CYP82G1* gene, involved in homoterpene synthesis, was significantly repressed in the shoots after RJA treatment, whereas TPS04 did not respond. These differential responses after RJA and SJA match previous studies showing that plants treated with JA to their shoots increased monoterpene, sesquiterpene and homoterpene emissions, whereas only monoterpene emissions increased when JA was applied to the roots [27]. Especially homoterpenes are important for attracting parasitic wasps and other natural enemies that play an important role in indirect defenses against herbivores [50], [51]. Indeed, behavioral experiments with herbivores and parasitoids showed that the differential metabolic response after root versus shoot induction, either with JA or real herbivores, had distinct effects on the parasitoid wasps associated with *Brassicaceae* [27], [28]. Parasitoids strongly preferred shoot induced over root induced plants. Volatile analysis demonstrated that root and shoot induced plants emitted increased levels of monoterpenes, but only shoot induced plants emitted enhanced levels of homoterpenes [27]. Because plant emitted volatiles play a key role in shaping the interactions of the plant with other organisms [5], the observed differential response in volatile synthesis depending on whether JA was applied to the roots of the shoots indicates that plants actively shape these interactions according to that organ that is wounded.

### JA Synthesis and Signaling

**Figure 6:**
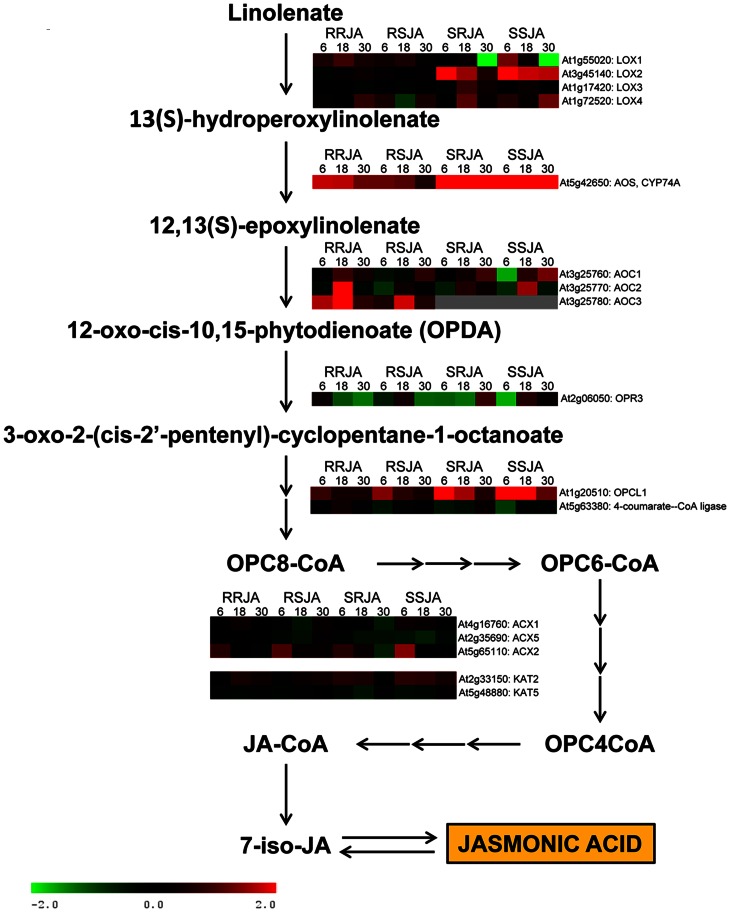
Gene expression in the JA synthesis pathway. In the shoots, a strong induction was observed of *LOX2* after JA treatment, whereas in the roots *LOX2* did not respond and only a two-fold up regulation of *LOX1* was observed. In both roots and shoots, RJA and SJA differentially affected the expression of the *AOC* and *OPR3* genes. Heat maps represent the log_2_ of the fold changes in expression after RJA or SJA compared to CON for all genes that were significantly affected in at least one of the treatment groups (ANOVA, FDR corrected *P*-value <0.1). RRJA, Root tissue RJA treatment; RSJA Root tissue SJA treatment; SRJA, Shoot tissue RJA treatment; SSJA, Shoot tissue SJA treatment.

In the hormone bin, two genes (*OPR3* and *AOC2*) encoding enzymes involved in JA synthesis showed a large differential response to RJA and SJA (Table S2). It is likely that the differentiation in the responses between SJA and RJA originate from the deviations early in the JA cascade or ensuing signaling processes. Therefore, we investigated the expression of genes involved in JA synthesis and signaling in more detail. In the shoots, there was a very strong up-regulation of *LOX2* and several other lox genes, whereas in the roots only *LOX1* was up-regulated two-fold at 18 h after RJA or SJA (Figure 6). The response of the genes more downstream in the pathway indicated that there was JA synthesis in the roots, but to a lesser extent than in the shoots, which corroborates earlier findings on JA responses after wounding or herbivory in maize [40]. Both artificial root damage and salt stress strongly induced MYC2, JAZ, JA-biosynthetic and defense-related gene expression in *A. thaliana* roots [52], [53]. Interestingly, we found a striking difference in the *OPR3* gene expression dynamics that depended on where JA was applied. In the roots, *OPR3* expression was significantly reduced at 18 and 30 h after RJA, but after SJA only at time point 30 h. In the shoots, RJA significantly reduced *OPR3* expression at time points 6 and 18 h, whereas SJA only reduced its expression at 6 h. Likewise, *AOC* (*Allene Oxide Cyclase*) gene expression differed depending on the site of JA application. In the roots, RJA significantly induced *AOC3* at 6 h and all 3 *AOC* genes at 18 h, while SJA only strongly induced *AOC2* at 18 h. AOC is involved in the synthesis of 12-oxophytodienoic acid (OPDA), and *OPR3* is responsible for the reduction of OPDA. The differential expression of both enzymes might have large effects on the concentration of OPDA itself, and probably also on the concentration of JA. Even though OPDA is a precursor in the synthesis of JA, the compound itself is also known to trigger a *COI1* independent defense response [54], [55], [56]. Moreover, JA and OPDA accumulation differ between organs: wounding of *A. thaliana* roots causes a JA and OPDA accumulation in the shoots at respectively 30 min and 6 h, while in roots JA and OPDA does not increase in concentration at these time points [52]. Therefore, a different OPDA/JA ratio depending on the site of JA induction might be one of the mechanisms causing the observed differential gene expression. To assess whether the differences in kinetics of JA and OPDA between organs play a role, the accumulation of both compounds should be measured after RJA and SJA. In conclusion, we found that the JA biosynthetic pathway is clearly differently regulated in roots and shoots, which may in turn cause the differential responses in both organs depending on where the initial JA signal was first perceived.

**Figure 7:**
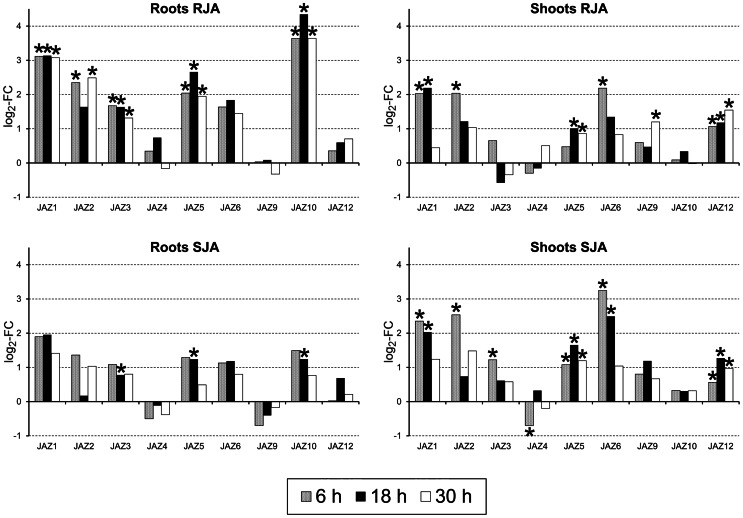
Differential expression of JAZ genes depending on tissue as well as site of induction. In the shoots, *JAZ1, 2, 5, 6* and *12* were induced after JA treatment, while in the roots mainly *JAZ1, 2, 3 5, 6* and *10* responded. In the roots, a 10-fold induction was found of *JAZ10* after RJA, while only a two-fold after SJA. Gene expression is shown as log_2_ of fold changes after RJA or SJA compared to CON. Samples in which the gene showed a significantly altered expression compared to CON are marked with an asterisk (ANOVA, FDR corrected *P*-value <0.1).

Another gene that showed a large difference in expression after RJA versus SJA was *JAZ10*, which is involved in transcriptional regulation of JA-induced gene expression (Table S2). In roots, the log_2_ fold change for *JAZ10* was about four after RJA, but only one after SJA at all three time points. JAZ proteins act as transcriptional repressors by binding to the MYC-2 transcription factor [9], [57], [58], [59]. Several other genes encoding different JAZ proteins were also significantly up-regulated by JA treatment (Figure 7). Interestingly, the expression profile of all these genes was strikingly different depending on the tissue as well as initial site of JA induction. In the shoots mainly *JAZ1, 2, 5, 6* and *12* were induced after JA treatment, while in roots mainly *JAZ1, 2, 3, 5, 6* and *10* responded. JAZ proteins are essential in the release of the transcription factor MYC2 from its repressors, resulting in the transcription of various JA-responsive genes [9], [60]. Degradation of a JAZ protein by the ubiquitin-proteasome pathway leads via a positive feedback loop towards transcriptional activation of its encoding gene [9]. Therefore, the profile of activated JAZ genes mimics the profile of the different degraded JAZ proteins. Moreover, the JAZ proteins function as homo- or heterodimers [9], [61], and most JAZ encoding genes in *A. thaliana* have several splice variants [62], thereby making the number of possible combinations of JAZ proteins even larger. It is unclear why so many different JAZ proteins are encoded in the genome [13]. Recently, several other transcription factors and co-repressors that interact with JAZ proteins were identified, among which several that are involved in the regulation of hormonal pathways other than JA [60]. It suggests that the large diversity of different JAZ proteins provides the plant with a mechanism to independently regulate separate parts of the elaborate JA signaling pathway. Therefore, the differential expression of the JAZ encoding genes indicates that already very early in the JA signaling pathway a distinct genetic program is activated depending on the tissue as well as the site of JA induction.

### Conclusions

By a transcriptomic and targeted metabolite analysis, we showed that both roots and shoots respond specifically to local and systemic induction with JA. A specific response depending on whether JA was applied to the roots or the leaves was found in primary metabolism (amino acids and carbohydrates) and some genes involved in regulation of plant development. Moreover, a root JA induction mainly induced aliphatic glucosinolate synthesis, while a leaf JA application resulted in an indole glucosinolate synthesis. Also the volatile synthesis was differentially influenced by a root or shoot JA application. Genes encoding enzymes involved in the synthesis of mono-, sesqui- and homoterpenes were induced after a shoot JA application, while only monoterpene biosynthesis genes were induced after a root JA induction. This indicates that plants not only adapt their growth and development, but also their defense response specifically to the organ that is induced. This raises questions about the nature of the systemic signal, which is not yet known. Artificial wounding of *A. thaliana* leaves causes local as well as distal accumulation of JA [11], [52]. Grafting experiments with different mutants demonstrate that the systemic response depends on JA synthesis at the site of wounding as well as on JA perception in the distal tissue [63]. However, it is as yet unclear whether JA, or JA conjugates, themselves serve as the systemic signals eliciting defense responses in undamaged plant organs [15]. Recent experiments suggest that the systemic wounding signal consists of a very fast transmembrane ion flux in the phloem, which might be followed by slower secondary signals [7], [59], [64], [65]. However, it is unlikely that transmembrane ion fluxes alone contain information about the initial induction site. We therefore hypothesize that the first quick signal consisting of transmembrane ion fluxes is followed by slower signals, which modify the JA-induced transcriptional program according to the site of initial induction. JA-conjugates are likely candidates conferring this information. Until now, more than 10 different JA-conjugates have been identified, including methyl esters and conjugates of different amino acids [11], [13]. Recently, transport of jasmonoyl isoleucine via the phloem after leaf wounding was demonstrated in tomato [66]. Transport of primary or secondary metabolites via the phloem may also play a role. Our observation that genes for extracellular export of sucrose in the roots were only induced after RJA and not after SJA supports this hypothesis. However, the basis for the differential gene expression between RJA and SJA is already evident in the early stages of JA signaling. Depending on the organ that was induced, a differential response was found for the JA biosynthesis and JAZ protein encoding genes. It suggests that the observed differential responses depending on the initial site of JA induction are not solely due to a simple reallocation of primary and secondary metabolites, but are the result of different JA signaling cascade in both organs. Independent of the exact nature of the systemic signal, our observations of a differential response in primary metabolism, development and defense depending on whether JA was applied to the roots or the shoots demonstrate that plants can make a distinction between signals coming from the roots or from the shoots. This enables them to fine-tune their responses specifically to the organ that has been damaged and has direct implications for the plant physiology as well as its interactions with other organisms. Further research is necessary to investigate the molecular mechanism behind this differential response and its effects on plant fitness and performance.

## Supporting Information

Figure S1 **To verify the microarray expression data, for five defense related genes a comparison was made between the gene expression levels measured by microarray analysis (right column) and by RT-qPCR (left column).** Although there were some small differences in the level of induction measured by RT-qPCR and microarray hybridization, the overall measured expression profiles were very similar. Expression is shown as fold changes compared to mock treatment at 6 h, 18 h and 30 h. Error bars represent the standard error of the mean.
(TIF)

Figure S2 **Amino acid concentrations were measured by HPLC in the roots after RJA, SJA and CON at day 1, 3, 7 and 14.** RJA resulted in a significant reduction in the concentration of glutamine at day 1 and (iso)leucine at day 1 and 3, whereas SJA caused a significant decrease of the concentration of (iso)leucine at day 1 and threonine at day 3. Concentrations are expressed in nmol/mg dry plant material after RJA (dotted line, open circles), SJA (dashed line, squares) or control treatment (solid line, triangles). Error bars represent the standard error. Treatments that resulted in a statistically significant different concentration compared to control are marked with an asterisk (p-value <0.05, t-test independent samples assuming unequal variances).
(TIF)

Figure S3 **Sugar concentrations were measured by HPLC in the roots after RJA, SJA and CON at day 1, 3, 7 and 14 after JA application.** RJA and SJA caused a decline in sucrose concentration compared to CON at day 1 and 3. Concentrations are expressed in nmol/mg dry plant material. RJA, dotted line and open circles; SJA, dashed line and squares; CON; solid line and triangles. Error bars represent the standard error. Samples with a significantly different concentration compared to control are marked with an asterisk (p-value <0.05, t-test independent samples assuming unequal variances).
(TIF)

Figure S4 **Gene expression in the Major CHO Metabolism bin in roots and shoots.** (a) In all treatments, sucrose synthesis was repressed. In roots, a strong induction of the genes for sucrose export across the plasma membrane was only observed after RJA. A clear induction of *Cytoplasmic Invertase 1* (*CINV1*) in roots after RJA, and to a lesser extent after SJA, indicates an increased degradation of sucrose into glucose and fructose. (b) In the shoots, a transient induction of genes involved in amylose synthesis and starch degradation at 6 h after RJA and SJA was observed, followed by strong repression of the latter thereafter. *Beta-amylase-5* (*Bam5*), involved in starch degradation into maltose, was strongly induced in the shoots at all time points after RJA and SJA, in the roots after SJA, and to a much lesser extent in the roots after RJA. For genes that were significantly differentially expressed in at least one of the treatment groups, a heat map is shown representing the log_2_ fold changes in expression compared to control treatment. RRJA, Root tissue RJA treatment; RSJA Root tissue SJA treatment, SRJA, Shoot tissue RJA treatment, SSJA, Shoot tissue SJA treatment.
(TIF)

Figure S5 **The differential response to RJA versus SJA is not due to a JA concentration effect.** Glucosinolate concentrations (+SE, n = 7 per treatment group, controls received equal amounts of acidic water pH = 3.7 applied to the roots and their shoots as their respective treatment groups) in leaves of *Brassica rapa* (Yellow Sarson) plants treated with increasing amounts of JA on the roots (RJA) or shoots (SJA). Glucosinolates were measured by HPLC on samples harvested seven days after treatment and grouped by biosynthetic origin: indole (black bars) and aliphatic glucosinolates (white bars). Letters over the bars indicate significant differences between treatment groups for indole (small letters) and aliphatic (capital letters) glucosinolate levels. MANOVA analysis revealed an overall significant treatment effect (F_4,102_ = 18.17, P<0.001), whereas JA concentration was not significant (F_4,102_ = 1.18, p = 0.32). The treatment×JA concentration effect was not significant either (F_4,102_ = 1.89, p = 0.07). Separate analysis of indole and aliphatic glucosinolates by ANOVA revealed similar patterns for each group. Combined with the results of the Tukey HSD analyses, this indicates that the JA response for both indole and aliphatic glucosinolates is saturated at 500 ug per plant. Moreover, the lack of response of the indole glucosinolates in RJA plants could not be alleviated by adding more JA to these plants.
(TIF)

Table S1 **Primer sequences used for RT-qPCR.**
(DOCX)

Table S2 **Expression of genes showing at least a 3-fold change after RJA versus SJA.**
(XLSX)
