# Correction: Plants Know Where It Hurts: Root and Shoot Jasmonic Acid Induction Elicit Differential Responses in *Brassica oleracea*

**DOI:** 10.1371/annotation/4574edb2-15bd-4689-bf84-b947ebe81ed2

**Published:** 2013-10-16

**Authors:** Tom O.G. Tytgat, Koen J. F. Verhoeven, Jeroen J. Jansen, Ciska E. Raaijmakers, Tanja Bakx-Schotman, Lauren M. McIntyre, Wim H. van der Putten, Arjen Biere, Nicole M. van Dam

A funding organization and two grants were incorrectly omitted from the Funding Statement. The Funding Statement should read: "T.O.G. Tytgat and N.M. van Dam were partly funded by a VIDI grant, no. 864.02.001, of the Netherlands Organisation for Scientific Research (NWO) to N.M. van Dam. K.J.F. Verhoeven was funded by a VENI grant, no. 863.05.006, of the Netherlands Organisation for Scientific Research (NWO). The funders had no role in study design, data collection and analysis, decision to publish, or preparation of the manuscript." 

