# Supplementary figures and images for: Plants Know Where It Hurts: Root and Shoot Jasmonic Acid Induction Elicit Differential Responses in Brassica oleracea
Source: PLoS One. 2013 Jun 11;8(6):e65502. doi: 10.1371/journal.pone.0065502 (PMC3679124; doi:10.1371/journal.pone.0065502)

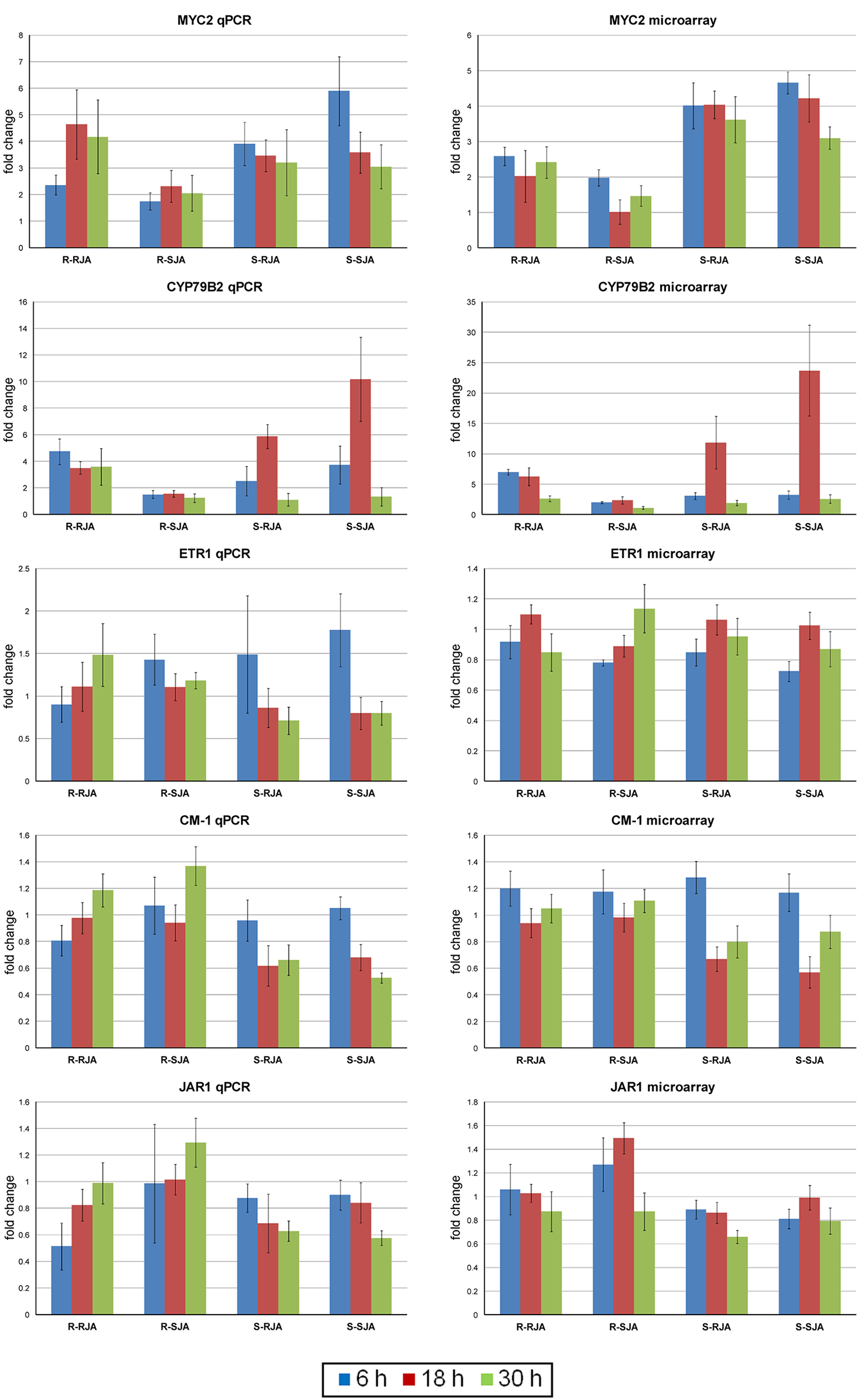

Supplement: Figure S1 — To verify the microarray expression data, for five defense related genes a comparison was made between the gene expression levels measured by microarray analysis (right column) and by RT-qPCR (left column). Although there were some small differences in the level of induction measured by RT-qPCR and microarray hybridization, the overall measured expression profiles were very similar. Expression is shown as fold changes compared to mock treatment at 6 h, 18 h and 30 h. Error bars represent the standard error of the mean. (TIF) [file pone.0065502.s001.tif]

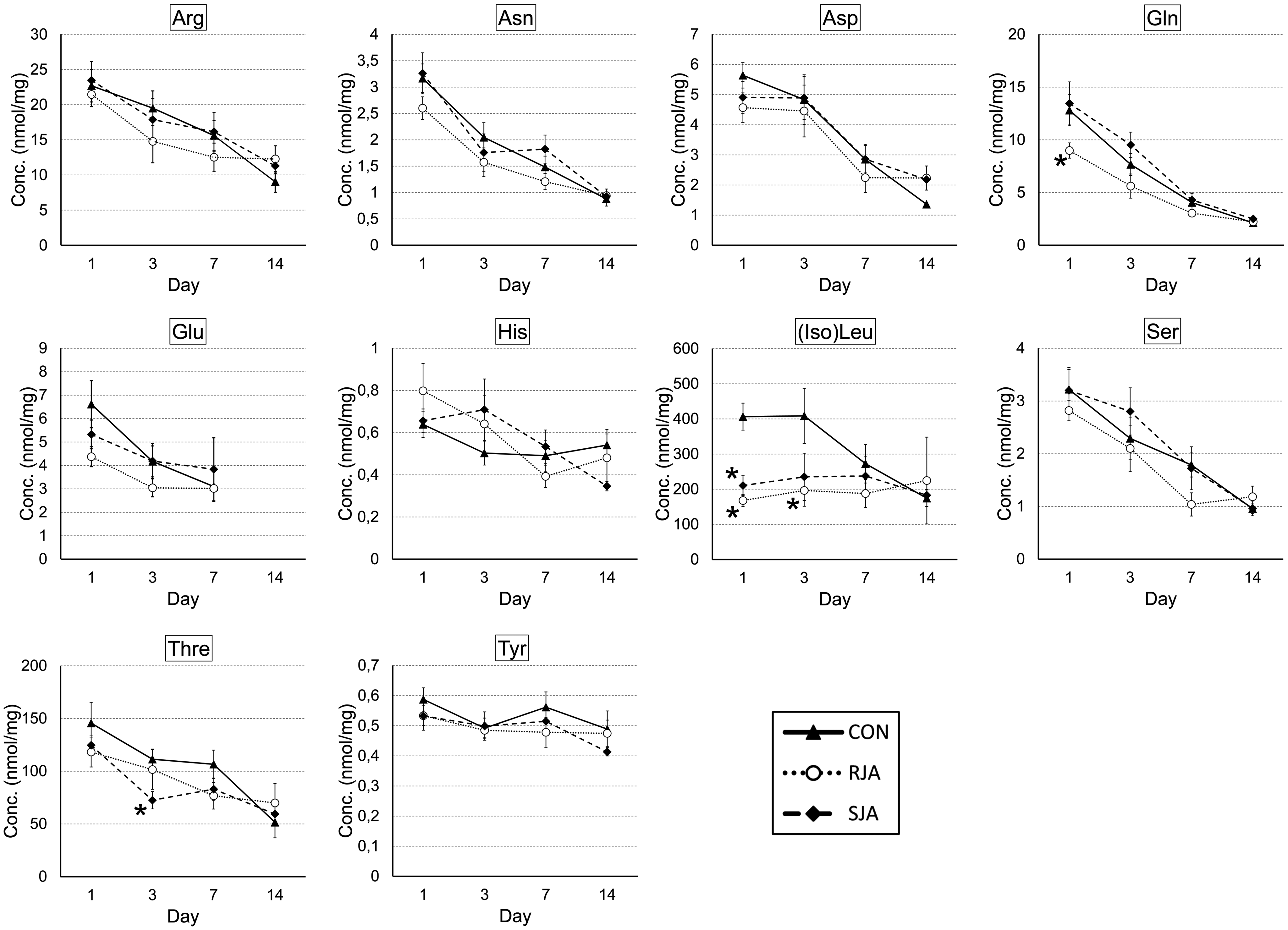

Supplement: Figure S2 — Amino acid concentrations were measured by HPLC in the roots after RJA, SJA and CON at day 1, 3, 7 and 14. RJA resulted in a significant reduction in the concentration of glutamine at day 1 and (iso)leucine at day 1 and 3, whereas SJA caused a significant decrease of the concentration of (iso)leucine at day 1 and threonine at day 3. Concentrations are expressed in nmol/mg dry plant material after RJA (dotted line, open circles), SJA (dashed line, squares) or control treatment (solid line, triangles). Error bars represent the standard error. Treatments that resulted in a statistically significant different concentration compared to control are marked with an asterisk (p-value <0.05, t-test independent samples assuming unequal variances). (TIF) [file pone.0065502.s002.tif]

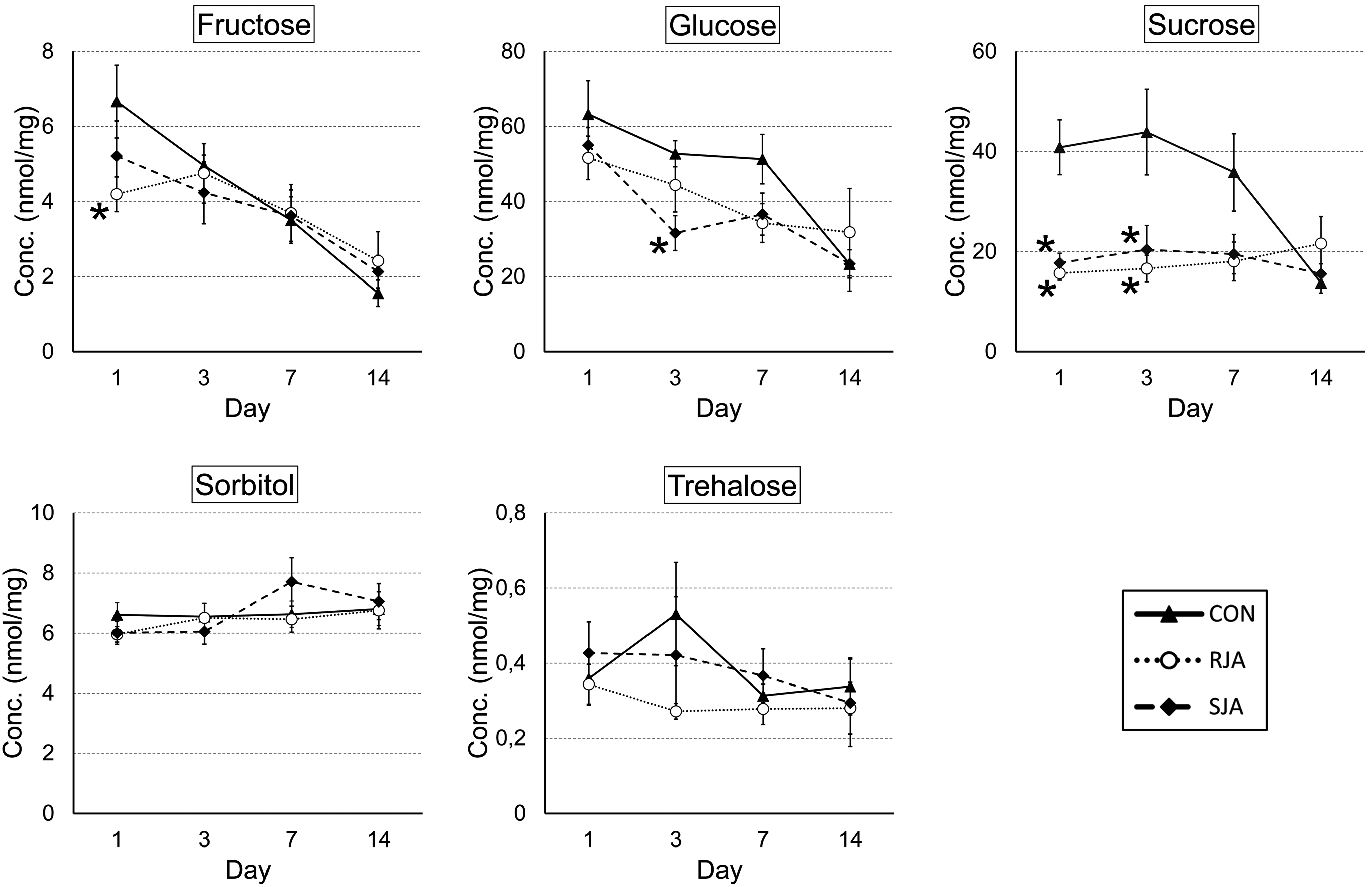

Supplement: Figure S3 — Sugar concentrations were measured by HPLC in the roots after RJA, SJA and CON at day 1, 3, 7 and 14 after JA application. RJA and SJA caused a decline in sucrose concentration compared to CON at day 1 and 3. Concentrations are expressed in nmol/mg dry plant material. RJA, dotted line and open circles; SJA, dashed line and squares; CON; solid line and triangles. Error bars represent the standard error. Samples with a significantly different concentration compared to control are marked with an asterisk (p-value <0.05, t-test independent samples assuming unequal variances). (TIF) [file pone.0065502.s003.tif]

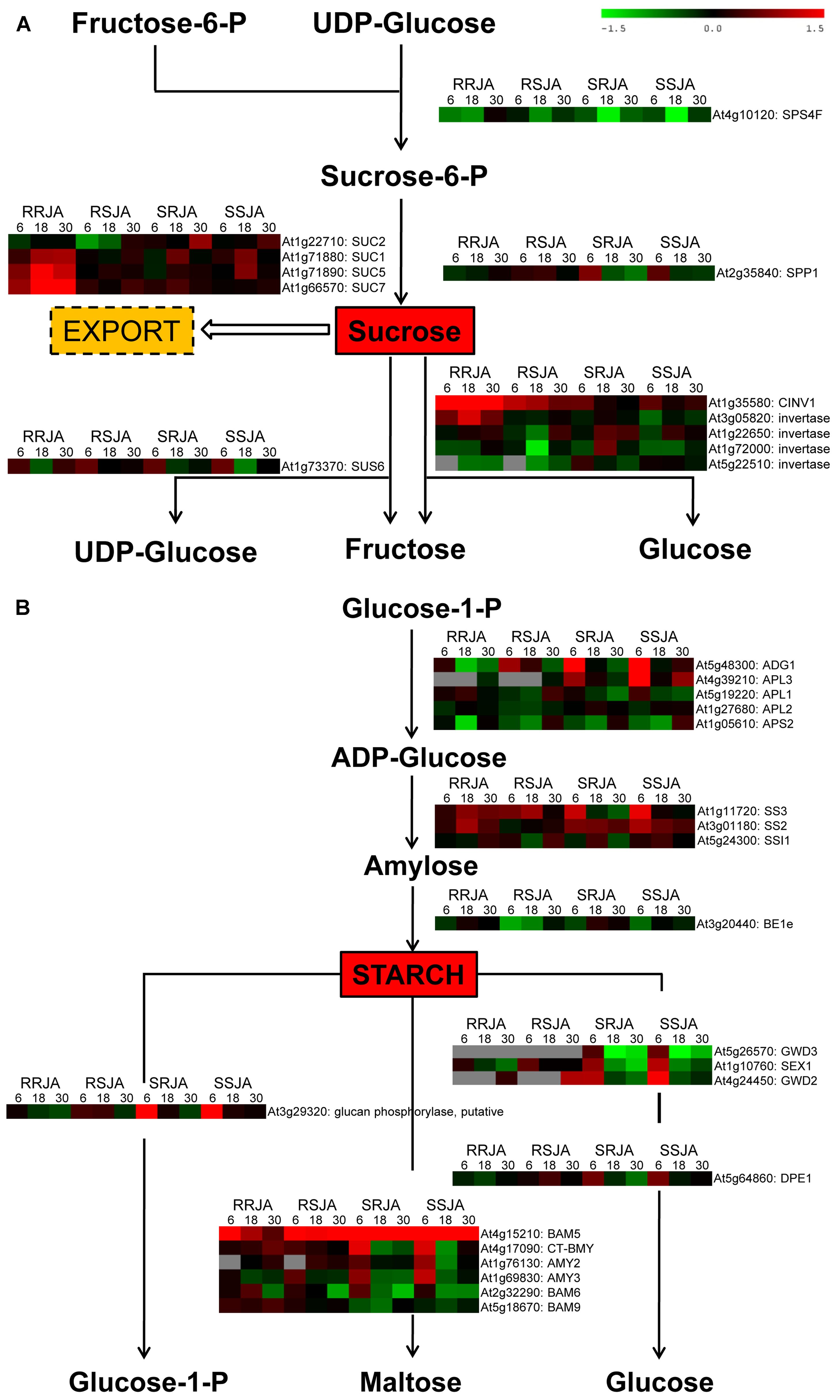

Supplement: Figure S4 — Gene expression in the Major CHO Metabolism bin in roots and shoots. (a) In all treatments, sucrose synthesis was repressed. In roots, a strong induction of the genes for sucrose export across the plasma membrane was only observed after RJA. A clear induction of Cytoplasmic Invertase 1 (CINV1) in roots after RJA, and to a lesser extent after SJA, indicates an increased degradation of sucrose into glucose and fructose. (b) In the shoots, a transient induction of genes involved in amylose synthesis and starch degradation at 6 h after RJA and SJA was observed, followed by strong repression of the latter thereafter. Beta-amylase-5 (Bam5), involved in starch degradation into maltose, was strongly induced in the shoots at all time points after RJA and SJA, in the roots after SJA, and to a much lesser extent in the roots after RJA. For genes that were significantly differentially expressed in at least one of the treatment groups, a heat map is shown representing the log2 fold changes in expression compared to control treatment. RRJA, Root tissue RJA treatment; RSJA Root tissue SJA treatment, SRJA, Shoot tissue RJA treatment, SSJA, Shoot tissue SJA treatment. (TIF) [file pone.0065502.s004.tif]

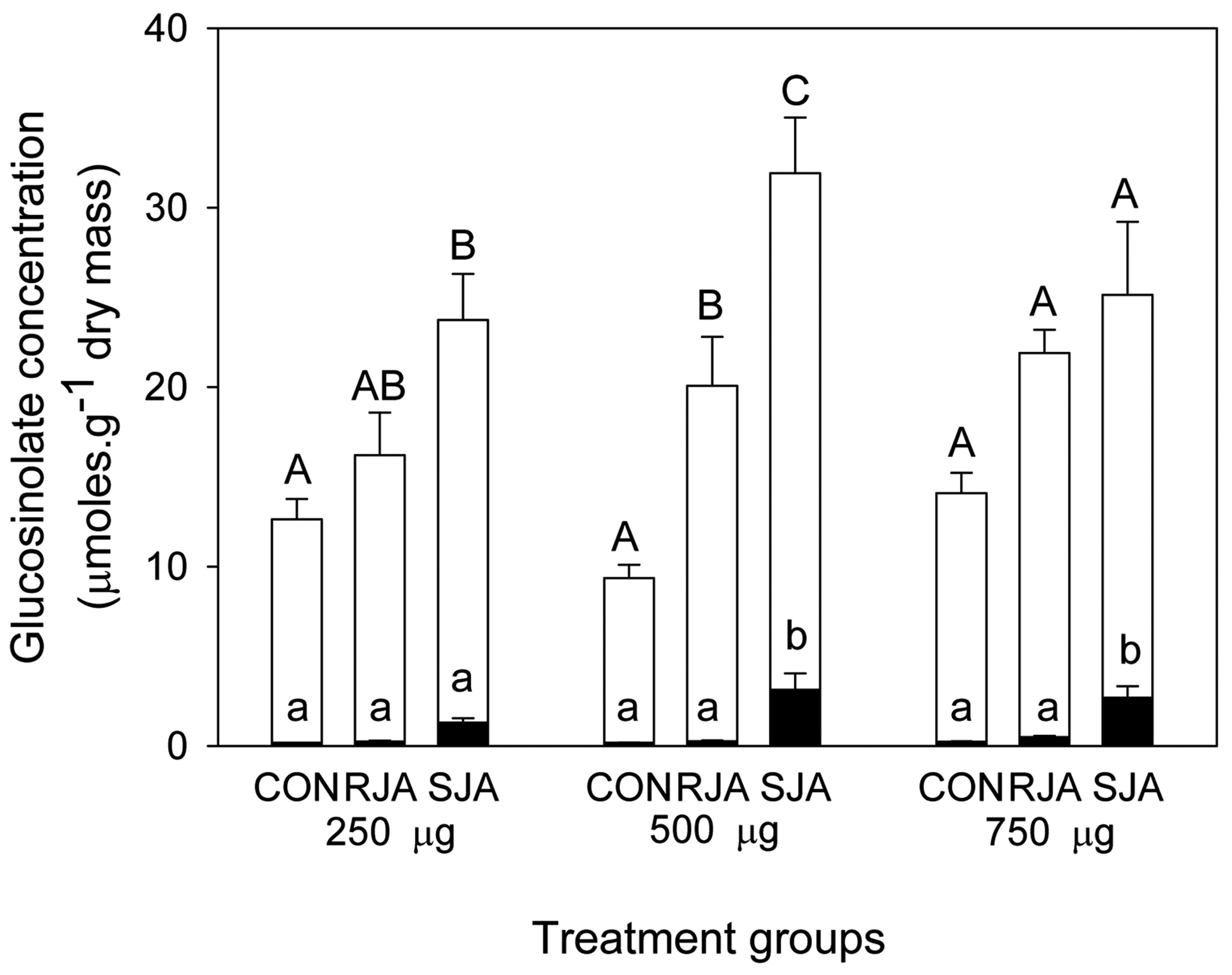

Supplement: Figure S5 — The differential response to RJA versus SJA is not due to a JA concentration effect. Glucosinolate concentrations (+SE, n = 7 per treatment group, controls received equal amounts of acidic water pH = 3.7 applied to the roots and their shoots as their respective treatment groups) in leaves of Brassica rapa (Yellow Sarson) plants treated with increasing amounts of JA on the roots (RJA) or shoots (SJA). Glucosinolates were measured by HPLC on samples harvested seven days after treatment and grouped by biosynthetic origin: indole (black bars) and aliphatic glucosinolates (white bars). Letters over the bars indicate significant differences between treatment groups for indole (small letters) and aliphatic (capital letters) glucosinolate levels. MANOVA analysis revealed an overall significant treatment effect (F4,102 = 18.17, P<0.001), whereas JA concentration was not significant (F4,102 = 1.18, p = 0.32). The treatment×JA concentration effect was not significant either (F4,102 = 1.89, p = 0.07). Separate analysis of indole and aliphatic glucosinolates by ANOVA revealed similar patterns for each group. Combined with the results of the Tukey HSD analyses, this indicates that the JA response for both indole and aliphatic glucosinolates is saturated at 500 ug per plant. Moreover, the lack of response of the indole glucosinolates in RJA plants could not be alleviated by adding more JA to these plants. (TIF) [file pone.0065502.s005.tif]
