# Supplementary material for: Plants Know Where It Hurts: Root and Shoot Jasmonic Acid Induction Elicit Differential Responses in Brassica oleracea
Source: PLoS One. 2013 Jun 11;8(6):e65502. doi: 10.1371/journal.pone.0065502 (PMC3679124; doi:10.1371/journal.pone.0065502)
Supplement: Table S1 — Primer sequences used for RT-qPCR. (DOCX) [file pone.0065502.s006.docx]

**Table S1** Primer sequences used for RT-qPCR

| **Gene** | **Annotation** | ***A. thaliana* orthologous AGI locus** | **Forward primer** | **Reverse primer** |
| --- | --- | --- | --- | --- |
| *GAPC2* | Glyceraldehyde-3-phosphate dehydrogenase C2 | At1G13440 | 5’-AGTTGTTGACCTCACGGTTAGAC-3’ | 5’-TTCCTCCTTGATAGCCTTCTTG-3’ |
| *PP2A* | Protein phosphatase 2A subunit A3 | At1G13320 | 5’-CATGCTCCAAGCTCTTACCTG-3’ | 5’-AATTTGATGTTTGGAACTCTGTCTT-3’ |
| *CYP79B2* | CYP79B2 monooxygenase | At4G39950 | 5’-AAGAGGTTGTGCTGCTCCG-3’ | 5’-TCCAAGTGAAACCTTGAAGAAGTC-3’ |
| *MYC2* | Myc2 transcription factor | At1G32640 | 5’-AGGTTGATGTCGGCGTTG-3’ | 5’-CGTTAACCACCGACATACTCG-3’ |
| *JAR1* | Jasmonate Resistant 1, jasmonate-amino synthetase | At2G46370 | 5’-CCAAGATGTGTGAAGCCAAG-3’ | 5’-CTCACCACATTCTCACACAGAA-3’ |
| *ETR1* | Ethylene Response 1, protein histidine kinase | At1G66340 | 5’-CGGTGAGTTCAAACGAGGAG-3’ | 5’-CACACGTCCATGAAGACCAC-3’ |
| *CM-1* | Chorismate Mutase 1 | At3G29200 | 5’-CTTCAGTCTCTTTCAAAGAGGATCC-3’ | 5’-CCTTATCTTGTGCTTTGATGGC-3’ |
